# Socioeconomic status, alcohol use and the role of social support and neighbourhood environment among individuals meeting criteria for a mental health problem: a cross-sectional study

**DOI:** 10.1007/s00127-024-02670-w

**Published:** 2024-04-26

**Authors:** Jo-Anne Puddephatt, Andrew Jones, Suzanne H. Gage, Laura Goodwin

**Affiliations:** 1 Department of Psychological Sciences, University of Liverpool, Liverpool, UK; 2 Division of Health Research, Lancaster University, Lancaster, UK; 3 Department of Psychology, Liverpool John Moores University, Liverpool, UK; 4 Department of Psychology, Edge Hill University, Ormskirk, UK

**Keywords:** Alcohol use, Mental health, Socioeconomic status, Social support, Neighbourhood environment

## Abstract

**Purpose:**

Indicators of socioeconomic status (SES), such as education and occupational grade, are known to be associated with alcohol use but this has not been examined among individuals with a mental health problem. This study developed latent classes of SES, their associations with alcohol use, and examined the indirect effect via social support and neighbourhood environment.

**Methods:**

A secondary analysis of the 2014 Adult Psychiatric Morbidity Survey was conducted among participants with a mental health problem (*N* = 1,436). SES classes were determined using a range of indicators. Alcohol use was measured using the Alcohol Use Disorder Identification Test. Social support and neighbourhood neighbourhood environment were measured using validated questionnaires. A latent class analysis was conducted to develop SES classes. Multinomial logistic regression examined associations of SES and alcohol use. Structural equation models tested indirect effects via social support and neighbourhood environment.

**Results:**

A four-class model of SES was best-fitting; “economically inactive,GCSE-level and lower educated,social renters”, “intermediate/routine occupation,GCSE-level educated,mixed owner/renters”, “retired, no formal education,homeowners”, and “professional occupation,degree-level educated,homeowners”. Compared to “professional occupation,degree-level educated, homeowners”, SES classes were more likely to be non-drinkers; odds were highest for “economically inactive,GCSE-level and lower educated,social renters” (OR = 4.96,95%CI 3.10–7.93). “Retired, no formal education,homeowners” were less likely to be hazardous drinkers (OR = 0.35,95%CI 0.20–0.59). Associations between “economically inactive,GCSE-level and lower educated,social renters” and “retired, no formal education,homeowners” and non- and harmful drinking via social support and neighbourhood environment were significant.

**Conclusions:**

In contrast to the alcohol harms paradox, among individuals with a mental health problem, lower SES groups were more likely to be non-drinkers while no associations with harmful drinking were found. There is also a need to examine the alcohol harms paradox in the context of the area in which they live.

**Supplementary Information:**

The online version contains supplementary material available at 10.1007/s00127-024-02670-w.

## Introduction

### Background

The global prevalence of past year alcohol use is 52.3%, while the prevalence of alcohol use disorder (AUD) is around 2.2% [1]. Alcohol is the most harmful drug when considering its impact on the individual and others [2]. Recent evidence has shown that the prevalence of AUD was higher among individuals meeting criteria for a mental health problem [3, 4], while mental health problems and AUDs contribute significantly towards disability-adjusted life years [5, 6] and mortality [7, 8].

The alcohol harms paradox [9] and social causation hypothesis [10, 11] suggest that individuals may be at greater risk of experiencing alcohol harms and reporting poorer mental health because of their socioeconomic status (SES). Previous research has shown that those of lower SES were more likely to experience alcohol harms, despite lower self-reported alcohol use [12–14]. Those from lower SES groups were also more likely to experience worsened mental health which may be explained by patterns of drinking [9, 12], other unhealthy behaviours [9], co-occurring health problems [9], and barriers to accessing services [9, 15]. A recent study also found that SES partially accounted for associations between alcohol use (including non-drinking), and mental health [16], suggesting that SES plays a role in this co-occurrence. However, SES is a multidimensional construct which comprises of different factors including economical resources and means, educational level, and occupation [17, 18], and previous research have used single measures of SES to examine associations with alcohol use [12] but these findings may differ depending on the measure of SES [13].

Latent class analysis (LCA) may be more appropriate because multiple measures of SES can be used to group individuals based on responding to different measures in a similar way. A previous study undertook a LCA which found that economically inactive (defined as being in receipt of benefits, being a renter, and having low education levels) groups were more likely to report harmful drinking; and having a common mental disorder (CMD) partially explained these increased associations in the general population [19]. Exploring SES in this way allows the researcher to examine the effect of having multiple advantages or disadvantages. Further, associations between SES and alcohol use may be stronger among individuals with poor mental health because they may experience multiple disadvantages.

There are also contextual factors which contribute towards the association between SES and different patterns of alcohol use among individuals who are experiencing mental health problems [16]. One factor is social support which can be defined by the social networks an individual has or by the type of support individuals receive from others which may come in the form of emotional (e.g. feeling loved), informational (e.g. providing information) or instrumental (e.g. tangible help) support [20]. The relationship between SES and social support is complex and its mechanisms are not well understood. It could be argued that individuals from lower SES backgrounds have more structural barriers and less opportunities to create a good level of social support [21, 22], for example, those from lower SES backgrounds may have less access to resources which could have a subsequent impact on accessing support, as such this could have an impact on health behaviours and outcomes. It could also be argued that social support acts as a buffer against stress and negative events [23]. Nonetheless, research has shown that social support can help to adjust to stressful conditions [24], while emotional support reduces the risk of a CMD [25]. The evidence on social support and drinking at harmful levels in the general population is mixed [26–28], but evidence suggests that social support can be particularly useful in maintaining non-drinking, particularly among those who have previously drank at harmful levels [29, 30]. However, despite research illustrating the associations between mental health and alcohol use [4, 16], there is seldom research which has explored the indirect role of social support on alcohol use among those with a mental health problem. Social support may be particularly beneficial for those with a mental health problem because of the additional stressors they may experience, however, to the author’s knowledge, this has not been explored. It could be argued that individuals from lower SES backgrounds with a mental health problem have less opportunities to create good quality social support, therefore have less recourses to cope and are more likely to use alcohol at harmful levels. This may explain previous research which has found associations between lower SES and harmful drinking after accounting for a CMD [19].

Another factor is the neighbourhood environment which is defined as the sense of belonging in the community, the presentation of surroundings, and the extent to which a person feels safe [31, 32]. Neighbourhood environment has been found to mediate relationships between SES with alcohol use [33–35] and mental health [31]. In the context of this study, it could be argued that individuals from higher SES backgrounds can afford to live in more advantaged areas where there is better access to mental health support and so alcohol may be used less to cope as indicated by the fundamental cause theory [36]. But little is known about how neighbourhood environment plays a role in the relationship between SES and alcohol use among individuals with a mental health problem.

This study aimed to address the following objectives among people meeting criteria for a mental health problem: i) understand how individuals are clustered based upon multiple indicators of SES to define latent classes, ii) determine associations between SES classes and alcohol use, and iii) examine whether there is an indirect effect of SES and alcohol use via social support and neighbourhood environment. We hypothesise that more advantaged SES classes will have increased odds of non-drinking or hazardous use and more disadvantaged classes will have increased odds of being a harmful/probable dependence. We also hypothesise that there will be an indirect association between more advantaged SES classes and non-drinking via increased social support and living in a better neighbourhood environment. Further, we hypothesise that there will be an indirect association between lower SES classes and harmful/probable dependence via decreased social support and living in a worse neighbourhood environment.

## Methods

### Study design

This study used data from the 2014 Adult Psychiatric Morbidity Survey (APMS) which uses a stratified multi-stage random probability sample. It is a cross-sectional survey of private households in England which has been on-going since 1993, with 2014 being the latest survey. 2014 APMS data was accessed through NHS Digital (ref. DARS-NIC-220105-B3Z3S-v0.3). We pre-registered our hypotheses and analyses (https://osf.io/h3ntf/).

### Participants and setting

A detailed description of the APMS methodology is described elsewhere [37]. One adult aged 16 or older was selected from each eligible household to take part in a face-to-face interview and reimbursed with a £15 high street voucher. Interviews were conducted in individuals’ homes and some information was collected by self-completion using computer-assisted interviewing. Interviews were conducted from May 2014 to September 2015 [37]. This study focused on those meeting criteria for a mental health problem which ranged from depression to probable psychotic disorder, further detail around the specific types of mental health problems included, measures and cut-offs used can be found in Table S1 and elsewhere [16].

### Measures

Given the multidimensional nature of SES, the following indices were used to measure SES within this study, each measure captures a different aspect of SES:

*Social occupational grade:* This variable was derived by combining responses from open questions on topics of the nature of the participants’ sector/industry, level of supervisory and managerial responsibilities as well as responses from the following items; ever having a job (“*Have you ever had a paid job, apart from casual or holiday work?*”) and reasons for not being in work (“*What was the main reason you did not seek any work in the last 4 weeks/would not be able to start in the next 2 weeks?*”). This variable was categorised as; i) managerial/professional, ii) intermediate, small employers and own account workers, iii) lower supervisory/technical/semi-routine/routine, iv) student, v) retired, and vi) never worked/not worked in the past year/not classified for other reason.

*In debt:* This variable was derived from item “*Have there been times during the past year when you or your household were seriously behind in paying within the time allowed for any of these items?*” and dichotomised as “yes” or “no”.

*In receipt of any out of work benefits:* This variable was derived from items “*Are you currently receiving any of these benefits as the named recipient*?” and *"Are you currently receiving any of these benefits either as the named recipient, or on behalf of someone in your household"* and dichotomised as “yes” or “no”.

*Highest education qualification* This variable was derived from item (“*Please look at this card and tell me whether you have passed any of the qualifications listed. Look down the list and tell me the first one you come to that you have passed.*”) and categorised as; i) University degree or higher, ii) A-Level/GCSE level, iii) other qualifications (including foreign qualifications), and iv) no qualifications.

*Housing tenure*:This variable was derived from two questions; “*In which of these ways do you (or your household) occupy this accommodation*?” and “*Who is your landlord*?”. Participants were categorised as; i) homeowner, ii) social renter, and iii) private renter.

*Household type:* Participants were asked to indicate their current household living situation and categorised as; i) lives alone, without children (reference), ii) lives with another adult without children, iii) lives in a family, and iv) lives in an adult household.

*Alcohol use:* Alcohol use was measured using two screening questions and participants’ scores on the Alcohol Use Disorder Identification Test (AUDIT); *“Do you ever drink alcohol nowadays?”*, those who responded “no” were then asked, *“Could I just check, does that mean you never have an alcohol drink nowadays, or do you have an alcoholic drink very occasionally?”*. Those who responded “no” did not complete the AUDIT. The AUDIT is a 10-item screen for alcohol use and related problems which has a maximum score of 40 [38], and good internal reliability within this sample (Cronbach’s α = 0.96). This tool has also been shown to have good validity [39]. Participants were categorised in accordance with established cut-offs [38]; “non-drinker” (answering “no” to screening questions or having an AUDIT score of 0), “low-risk” (AUDIT score:1–7, reference), “hazardous use” (AUDIT score:8–15), and “harmful/probable dependence” (AUDIT score:16 or above).

*Social support:* Perceived social support was measured using a seven-item questionnaire from the 1987 Health and Lifestyle Survey and has a maximum score of 21 [40]. The questionnaire is publicly accessible [41] and includes statements that individuals responded to as “not true”, “partly true”, or “certainly true” of their family and friends. These statements include the extent to which the participant believes that their friends and family i) do things to make them happy, ii) make them feel loved, iii) can be relied upon, iv) ensure that they would be taken care of, v) accept them as they are, vi) make them feel important, vii) give them support and encouragement. This measure has good internal reliability (Cronbach’s α = 0.94) and its validity has been established elsewhere [42, 43]. This measure was treated as a continuous variable, with a high score indicating strong social support.

*Neighbourhood environmen:t* Neighbourhood environment was measured using a 10-item questionnaire which asks participants questions around social cohesion [44] and neighbourhood quality [45]. All items except one (“*The area is kept nice by its residents”*) had good item-test correlation with the proposed constructs (Cronbach’s inter-item test correlation = 0.73–0.89). The item with lower item-test correlation compared to other items in the questionnaire (Cronbach’s inter-item test correlation = 0.42) was removed from analysis, thus, a nine-item questionnaire was used to generate a total score (Cronbach’s α = 0.84, RMSEA = 0.07, CFI = 0.98) with a maximum score of 45. This measure was treated as a continuous variable, with a high score indicating worse neighbourhood environment.

### Sample size

The full sample size of the 2014 APMS was 7546, but after restricting to those individuals who met criteria for a mental health problem, the final sample was 1436.

### Statistical analysis

**Fig. 1:**
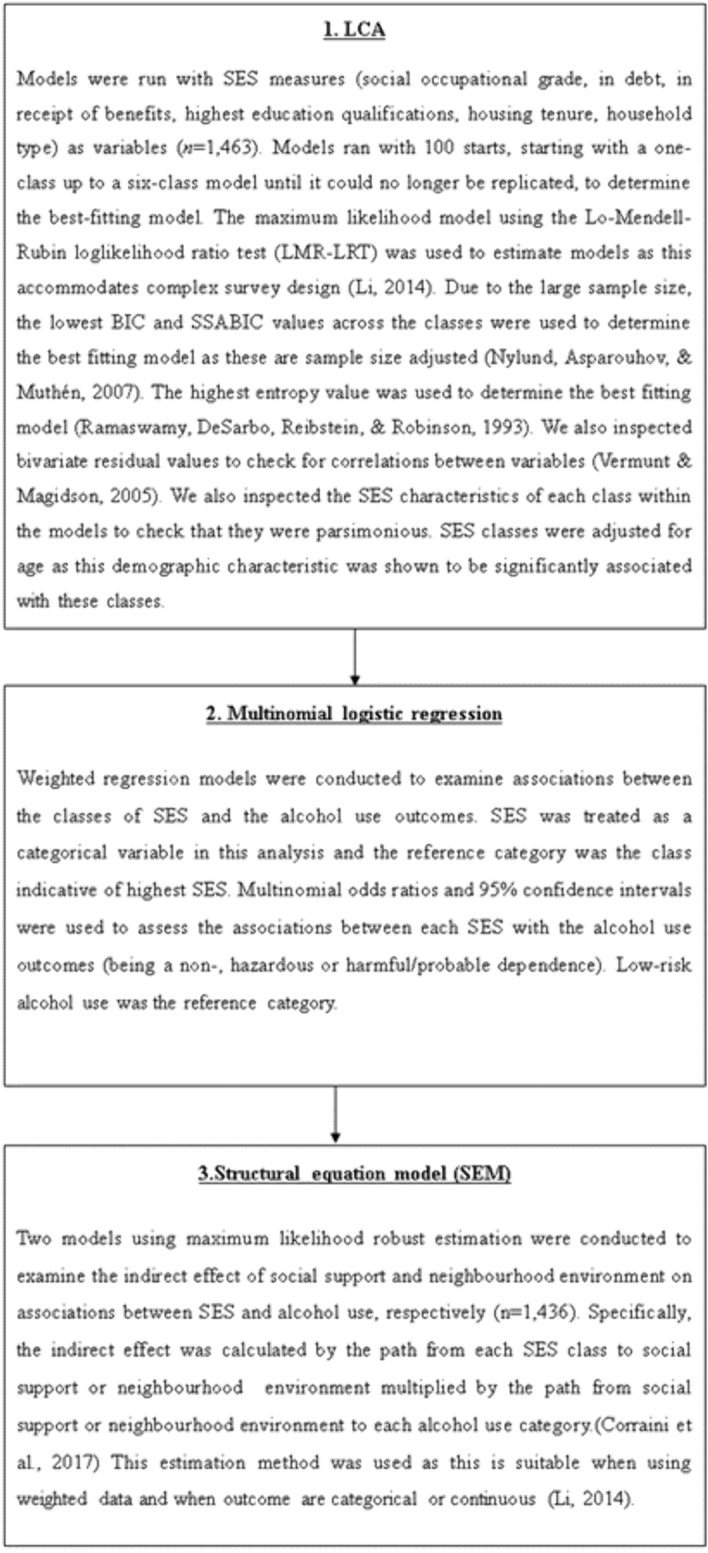
A flow chart of the data analysis strategy

Data was processed in STATA version 16 and analysed in MPlus version 8. A previous study using this dataset reported that most variables assessing specific types of mental health problems had a small amount of missing data (~ 0.01%) though alcohol use had 3.7% of missing data [16]. There was also a small amount of missing data for SES measures, social support and neighbourhood environment (1.03–1.64%). Of those included in the survey (*N* = *1*,*463*), 27 (2.51%) did not complete the alcohol measures, therefore, a case-complete analysis was conducted. Potential non-response could be due to items being in the self-completion section of the questionnaire [37]. The analysis took part in three stages (see Fig. 1):

Indirect effects of social support and neighbourhood environment were calculated using the “MODEL CONSTRAINT” command where the path from each SES class to social support or neighbourhood environment were multiplied with the paths from social support or neighbourhood environment to each alcohol use category [46]). This allowed us to understand whether the indirect effect of social support and neighbourhood environment partially or fully accounted for the association between each SES class and alcohol use outcome [47]. Regression coefficients for indirect paths with continuous outcomes (social support and neighbourhood environment) were unstandardized and calculated using the Delta method [48]. Multinomial logistic regression coefficients for indirect paths with categorical outcomes (non-drinking, hazardous drinking, and harmful/probable dependence) were reported. To enable comparisons between the associations between each SES class and alcohol use outcomes, the exponentiated multinomial logit coefficients are reported in the figures. Due to the dependent variable being nominal, it was not possible to use additional model fit indices to assess the indirect associations of SES and alcohol use.

Multinomial logistic regression was used to calculate the paths from SES to alcohol use, and paths from social support and neighbourhood environment to alcohol use, respectively. For the multinomial logistic regressions in these models, the multinomial odds ratio, confidence intervals not overlapping with 1 and a p-value of less than 0.05 was deemed statistically significant. All analyses were conducted in MPlus version 8 and weighted to account for selection probabilities and non-response.

### Sensitivity analysis

A sensitivity analysis of the prevalence of SES classes among individuals who met criteria for a CMD (defined as depressive and anxiety disorders [49]) or severe mental illness (SMI; defined as bipolar disorder, probable psychotic disorder and any other psychotic disorder [50]) was conducted. Household income was added to the latent class model as part of an additional sensitivity analysis. Household income was a categorical variable (less than £12,999, more than or equal to £12,999 or less than £20,279, more than or equal to £20,279 or less than £31,666, more than or equal to £31,666 or less than £52,499, more than or equal to £52,499) and there was 20.98% of missing data for this variable. Values were imputed through multiple imputation by chained equations [51] in STATA version 16. The imputation model included all of the SES variables used in the final latent class analysis (social occupational grade, being in debt, being in receipt of any out of work benefits, educational attainment, housing tenure and household type) and alcohol use were included in the imputation model which is considered as best practice for multiple imputation [52]. Ten cycles of the imputations were run. Once data was imputed, data were re-analysed as per steps outlined above. Analyses using the imputed and non-imputed data showed similar results, therefore, non-imputed data are shown in the results tables (see Table 2 and 3) while imputed data are shown in supplementary (see Tables s6-s10).

## Results

### Latent class solutions

Table S3 shows the model fit indices of a one- to six-class model of SES among those who met criteria for a mental health problem. A four and five-class model indicated good fit based on these having lower AIC, BIC and SSABIC values, while also having a high entropy value. The biggest drop in AIC BIC and SSABIC values were observed from a three to four-class model and further inspection of the classes between the four and five-class models suggested that the additional class was mainly defined by lower supervisory occupations and thus less parsimonious.

### Descriptions of four-class model

Table S4 shows the probability of being assigned to each of the four classes based on individual SES indicators. An overview of each class is provided below. A sensitivity analysis showed that 36.03% of those with a CMD were “routine/intermediate occupation, GCSE-level educated, mixed owner/renters” whereas 18.94% were “retired, no formal educated, homeowners”. Of those with a SMI, 42.87% were “economically inactive, GCSE-level and lower educated, social renters” whereas 8.56% were “retired, no formal educated, homeowners” (Table S4).

#### Class one—“Economically inactive, GCSE-level and lower educated, social renters” (N = 341, 19.25%), probability of correct identification = 88.0%)

There were approximately an equal number of males and females in this class, with half aged 35–54, of White ethnicity, and half single (Table 1). The majority of this group (80.00%) were not working/have not worked in the past year, 42.00% in debt and 84.00% in receipt of any out of work benefits. While 46.00% were educated to A-Level or GCSE level and 43.00% had no educational qualifications. Regarding housing tenure, 64.00% of this group were social renters and more likely to live in a household with another adult, without children, compared to living alone, without children (Table S4).

**Table 1:** Demographic characteristics of latent classes of SES

Demographic characteristics	Class one: “Economically inactive, GCSE-level and lower educated, social renters” (n = 341, 19.25%)	Class two: “Routine/intermediate occupation, GCSE-level educated, mixed owner/renters” (n = 440, 42.24%)	Class three: “Retired, no formal education, homeowners” (n = 311, 16.31%)	Class four: “Professional occupation, degree-level educated, homeowners” (n = 339, 22.20%)
	n (weighted %)	n (weighted %)	n (weighted %)	n (weighted %)
*Gender*				
Male	146 (52.80)	171 (52.61)	107 (37.22)	137 (49.82)
Female	195 (47.20)	269 (47.39)	204 (62.78)	202 (50.18)
*Age*				
16–34	93 (30.60)	228 (66.52)	–	92 (33.33)
35–54	173 (51.32)	202 (32.07)	–	186 (51.27)
55–74	75 (18.08)	10 (1.41)	228 (73.65)	61 (15.41)
75 +	–	–	83 (26.35)	–
*Ethnicity*				
White	304 (87.88)	377 (81.73)	300 (95.31)	293 (86.80)
Non-white	36 (12.12)	63 (18.27)	11 (4.69)	45 (13.20)
*Marital status*				
Single	188 (53.91)	239 (65.01)	31 (7.07)	127 (38.71)
Married or in civil partnership	49 (20.24)	137 (27.51)	143 (59.21)	148 (49.61)
Separated/divorced/widowed	104 (25.85)	64 (7.48)	137 (33.72)	64 (11.68)

#### Class two—“Routine/intermediate occupation, GCSE-level educated, mixed owner/renters” (N = 440, 42.24%), probability of correct identification = 93.30%)

There were an equal number of males and females in this class, two thirds were aged 16–34, the majority of White ethnicity, and two thirds single (Table 1). The majority of this group were in routine or intermediate occupations (66.00%) with 84.00% not in debt and 96.00% not in receipt of any out of work benefits. While 72.00% were educated to A-Level or GCSE level, 46.00% were homeowners and, also more likely to live in a household on their own, without children (Table S4).

#### Class three—“Retired, no formal education, homeowners” (N = 311, 16.31%), probability of correct identification = 87.60%)

Nearly two-thirds of this group were female, all aged 55 or over, of White ethnicity, and half married or in a civil partnership (Table 1). Most were retired (76.00%) and homeowners (72.00%). The majority were not in debt (95.00%) and 95.00% were not in receipt of any out of work benefits. Within this group, 44.00% had no qualifications and more likely to live in a family household, compared to living on their own, without children (Table S4).

#### Class four—Professional occupation, degree-level educated, homeowners” (N = 339, 22.20%), probability of correct identification = 92.00%)

There were an equal proportion of males and females in this class, half aged 35–54, the majority of White ethnicity and half married or in a civil partnership (Table 1). Most were in managerial or professional occupations (76.00%), 95.00% were not in debt and 99.00% were not in receipt of any out of work benefits. Sixty-seven percent were educated to degree level while 70.00% were homeowners. This group were more likely to live in a family household, compared to living alone, without children (Table S4).

### Associations of SES with alcohol use

Table 2 shows the frequency and associations of the SES classes with non-drinking, hazardous and harmful/probable dependence. The highest prevalence of non-drinking was among “economically inactive, GCSE-level or lower educated, social renters” and “retired, no formal education, homeowners” (35.94% and 34.99%, respectively) whereas the lowest prevalence was among “professional occupation, degree-level educated, homeowners” (10.23%). Over 25% of “routine/intermediate occupation, GCSE-level educated, mixed owner/renters” and “professional occupation, degree-level educated homeowners” reported hazardous use compared to 14.65% of “economically inactive, GCSE-level or lower educated, social renters” and 9.09% “retired, no formal education, homeowners”. Whereas 10.99% of “economically inactive, GCSE-level or lower educated, social renters” reported harmful/probable dependence compared to 3.87% of “retired, no formal education, homeowners” but proportions were similar between “routine/intermediate occupation, GCSE-level educated, mixed owner/renters” and “professional occupation, degree-level educated homeowners”.

**Table 2:** Weighted associations of classes of SES and alcohol use

	Non-drinker			Low-risk use (reference group)	Hazardous use			Harmful/probable dependence		
	*n* (weighted %)	MOR (95% CI)	*p*	*n* (weighted %)	*n* (weighted %)	MOR (95% CI)	*p*	*n* (weighted %)	MOR (95% CI)	*p*
Class 1—Economically inactive, GCSE-level or lower educated, social renters	119 (35.94)	**4.96 (3.10–7.93)**	**0.01**	118 (38.42)	51 (14.65)	0.76 (0.47–1.21)	0.28	33 (10.99)	1.90 (0.99–3.64)	0.08
Class 2—Routine/intermediate occupation, GCSE-level educated, mixed owner/renters	91 (21.61)	**2.56 (1.62–4.06)**	**0.01**	208 (44.69)	186 (25.55)	1.13 (0.75–1.70)	0.57	36 (8.15)	1.21 (0.65–2.27)	0.55
Class 3**—**Retired, no formal education, homeowner	101 (34.99)	**3.57 (2.24–5.67)**	**0.01**	147 (52.05)	27 (9.09)	**0.35 (0.20–0.59)**	**0.01**	12 (3.87)	0.49 (0.23–1.07)	0.11
Class 4—Professional occupation, degree-level educated, homeowners	40 (10.23)	Ref.	Ref.	186 (54.23)	81 (27.37)	Ref.	Ref.	25 (8.18)	Ref.	Ref.

^*^Bold indicates statistical significance (p < .05)

*MOR* multinomial odds ratio, *CI* confidence interval

Compared to “professional occupation, degree-level educated, homeowners”, all other SES classes had increased odds of non-drinking with associations strongest among “economically inactive, GCSE-level or lower educated, social renters” (OR = 4.96, 95%CI 3.10–7.93, see Table 2), only “retired, no formal education, homeowners” had decreased odds of hazardous use (OR 0.35, 95%CI 0.20–0.59, see Table 2). No associations between SES classes and harmful/probable dependence were found (see Table 2).

### Indirect effect of associations between SES and alcohol use via social support and neighbourhood environment

#### Social support

**Fig. 2:**
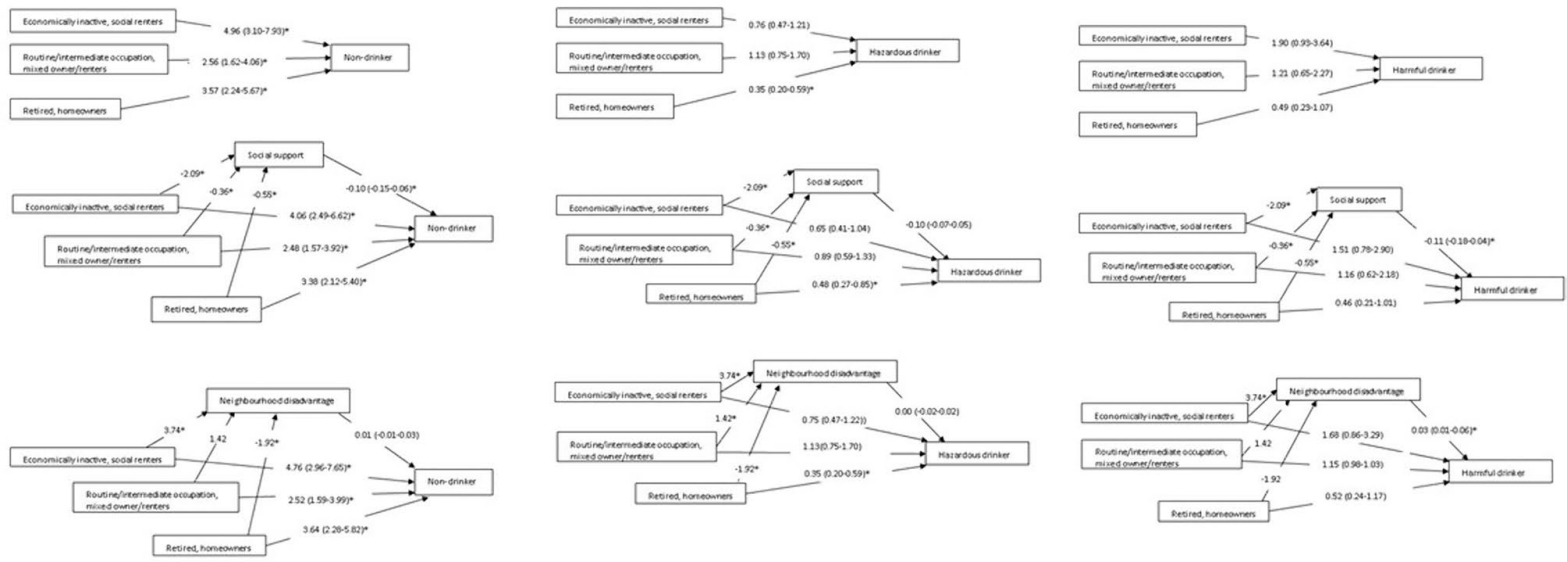
The indirect associations of each SES class and alcohol use via social support and neighbourhood environment, *The paths from each SES class to social support or neighbourhood environment are unstandardized coefficients. The paths from social support or neighbourhood environment to alcohol use are multinomial logit regression coefficients. The paths from each SES class to alcohol use are exponentiated multinomial logit regression coefficients

The mean social support score was 19.25 (SD = 3.02), indicating moderate levels of social support. Compared to “Professional occupation, degree-level educated, homeowners”, all other SES classes reported significantly lower social support scores (see Fig. 2). A higher social support score (compared to lower) was associated with a decreased odds of non-drinking and harmful/probable dependence, respectively (see Fig. 2). Associations between SES and non-drinking via social support were significant among “economically inactive, GCSE-level or lower educated, social renters” and “retired, no formal education, homeowners” (see Table 3), indicating that lower social support facilitated non-drinking among these groups. Whereas associations between “economically inactive, GCSE-level or lower educated, social renters” and harmful/probable dependence via social support was significant which suggests that lower social support also facilitated lower odds of harmful/probable dependence among this group.

**Table 3:** The indirect effect of associations between SES and alcohol use via social support and neighbourhood environment

		Social support (*n* = 1,436)			Neighbourhood environment (*n* = 1,436)		
Class 1—Economically inactive, GCSE-level or lower educated, social renters		**Unstandardised coefficient (SE)**	**95% CI**	***p***	**Unstandardised coefficient (SE)**	**95% CI**	***p***
	Non-drinker	**0.21 (0.06)**	**0.11–0.31**	**0.01**	0.04 (0.04)	−0.03–0.11	0.33
	Low-risk drinker	Ref.	Ref.	Ref.	Ref.	Ref.	Ref.
	Hazardous drinker	0.02 (0.07)	−0.09–0.14	0.76	0.00 (0.05)	−0.08–0.08	0.94
	Harmful/probable dependent drinker	**0.23 (0.09)**	**0.08–0.39**	**0.01**	**0.13 (0.06)**	**0.03–0.22**	**0.03**
Class 2—Routine/intermediate occupation, GCSE-level educated, mixed owner/renters		**Unstandardised coefficient (SE)**	**95% CI**	***p***	**Unstandardised coefficient (SE)**	**95% CI**	***p***
	Non-drinker	0.04 (0.02)	0.00–0.07	0.07	0.02 (0.02)	−0.01–0.05	0.37
	Low-risk use	Ref.	Ref.	Ref.	Ref.	Ref.	Ref.
	Hazardous use	0.00 (0.01)	−0.02–0.02	0.76	0.00 (0.02)	−0.03–0.03	0.94
	Harmful/probable dependence	0.04 (0.02)	0.00–0.08	0.09	0.05 (0.03)	0.00–0.09	0.08
Class 3—Retired, no formal education, homeowners		**Unstandardised coefficient (SE)**	**95% CI**	***p***	**Unstandardised coefficient (SE)**	**95% CI**	***p***
	Non-drinker	**0.06 (0.02)**	**0.02–0.09**	**0.01**	−0.02 (0.02)	−0.06–0.02	0.36
	Low-risk use	Ref.	Ref.	Ref.	Ref.	Ref.	Ref.
	Hazardous use	0.01 (0.02)	−0.03–0.04	0.76	−0.00 (0.02)	−0.04–0.04	0.94
	Harmful/probable dependence	**0.06 (0.03)**	**0.01–0.11**	**0.04**	-0.06 (0.03)	−0.12–−0.01	0.06
Class 4—Professional occupation, degree-level educated, homeowners		Ref.	Ref.	Ref.	Ref.	Ref.	Ref.

*SE* standard error, *CI* confidence intervals

^*^Bold indicates significance

#### Neighbourhood environment

The mean neighbourhood environment score was 20.63 (SD = 7.42), indicating moderate levels of disadvantage. Compared to “professional occupations, degree-level educated, homeowners”, “economically inactive, GCSE-level or lower educated, social renters” reported higher neighbourhood environment scores whereas “retired, no formal education, homeowners” reported lower scores (see Fig. 2). An increase in neighbourhood environment was associated with increased odds of harmful/probable dependence, compared to low-risk drinking (see Fig. 2). Associations between “economically inactive, GCSE-level or lower educated, social renters” and harmful/probable dependence via neighbourhood environment was significant (see Table 3), indicating that among lower SES groups with a mental health problem, living in a worse neighbourhood environment played a role in the likelihood of harmful drinking.

## Discussion

### Key findings

This study aimed to examine SES classes of individuals who met criteria for a mental health problem, how SES was associated with alcohol use within this sample and examine the indirect effects via social support and neighbourhood environment. Four classes of SES were identified; “economically inactive, GCSE-level or lower educated, social renters”, “routine/intermediate occupation, GCSE-level educated, mixed owner/renters”, “retired, no formal education, homeowners”, and “professional occupation, degree-level educated, homeowners”, which occupation, GCSE-level educated, mixed owner/renters”. Compared to “professional occupation, degree-level educated, homeowners”, all other SES classes reported moderate to strong associations with non-drinking, while “retired, no formal education, homeowners” also reported decreased odds of hazardous use. The study found some evidence of an indirect effect of social support and neighbourhood environment with associations between “economically inactive, GCSE-level or lower educated, social renters” and non-drinking, and harmful/probable dependence, respectively.

### Links with previous research

SES classes among individuals who met criteria for a mental health problem reported in this study indicates that those with a mental health problem presented from a range of SES backgrounds which is in contrast to the social causation hypothesis where individuals report poor mental health because of their SES [10]. Our findings may be explained by the broad scope of mental health problems as we found that the prevalence of “economically inactive, GCSE-level educated, social renters” was highest among those who met criteria for a SMI and this is consistent with some previous research on lower individual and parental SES and more severe mental health problems [53, 54].

We hypothesised that more advantaged SES classes would have increased odds of non-drinking and decreased odds of harmful drinking, however, compared to “professional occupation, degree-level educated, homeowners”, we found that all other lower SES classes were more likely to report non-drinking. These findings may be explained by the “sick-quitter” hypothesis which suggests that individuals no longer drink because they were a previous harmful drinker [55] or have a pre-existing condition [56], which in the context of this study was having a mental health problem. However, it was not possible to explore the reasons for non-drinking in this sample.

We hypothesised that more disadvantaged SES classes would have increased odds of harmful/probable dependence, however, no associations were found, compared to “professional occupation, degree-level educated, homeowners” among individuals who met criteria for a mental health problem. The alcohol harm paradox argues that those of lower SES are more likely to experience alcohol harms despite drinking at lower levels [9] which has been consistently supported by previous research conducted in the general population [12, 14]. A recent study, which used LCA to derive SES, found higher levels of harmful drinking in “economically inactive homeowners” and “professional renters” compared to “professional homeowners” respectively in the general population [19]. While this contradicts our current findings, both showed that occupational grade and housing tenure were associated with alcohol use but there may be differences in how these were associated due to our sample being restricted to those who met criteria for a mental health problem, and the potential for the lack of associations with harmful/probable dependence being explained by participants being former harmful drinkers. While both the current study and Boniface and colleagues study [19] are limited by the cross-sectional design of the data, a review of the associations between SES and alcohol use has indicated that housing status is a predictor of alcohol use and negative alcohol-related consequences within the general population [57]. The longitudinal association between SES, alcohol use and social support has been seldom explored among individuals with a mental health problem. Our findings highlight the need for further research examining the longitudinal associations between SES and alcohol use (including non-drinking) among those with a mental health problem, particularly among the most deprived groups.

Some of the associations between SES and alcohol use were partially explained by lower social support and living in a worse neighbourhood environment, specifically the increased odds of non-drinking in the “economically inactive, GCSE-level or lower educated, social renters”, and harmful/probable dependence, and “retired, no formal education, homeowners” and non-drinking respectively. It can be argued that those from lower SES backgrounds have more barriers and less opportunities to create social support [21, 22] or that social support acts as a buffer against the use of alcohol when under stress [23]. Findings from this study suggest that decreased social support increased the odds of non-drinking and harmful/probable dependence, respectively. It may be the type of social support an individual has which might explain the significant findings to two different drinking patterns [24, 25].

In addition, when neighbourhood environment was considered, associations between SES and harmful/probable dependence was partially explained by living in a worse neighbourhood environment, specifically in partially explaining the increased odds of harmful/probable dependence in the “economically inactive, GCSE-level or lower educated, social renters”. While some of our findings contrast the social causation hypothesis [10], the indirect effect of neighbourhood environment indicate that the most deprived SES groups living in the most deprived areas may use alcohol to cope. Previous research has also shown an increase in alcohol consumption among men living in disadvantaged neighbourhoods [58], and an increase in severe hazardous drinking among women living in disordered neighbourhoods [59].

### Strengths and limitations

The current study is one of the first to show differences in associations among individuals who met criteria for a mental health problem which contrasts with findings from general population samples. We used a range of measures to capture different aspects of SES to develop a more holistic understanding of SES among this population. We also showed that social contexts, such as social support and neighbourhood environment, play a role in the associations between SES and alcohol use, which can be used to provide more tailored interventions towards the most deprived SES groups.

However, while individuals who met criteria for a mental health problem presented from a range of SES backgrounds, there may have been differences in these classes based upon the severity of the mental health problem as indicated by our sensitivity analysis, but we did not have enough statistical power to examine this in our multinomial regression and structural equation models. Further, the data used in this study was collected in 2014–2015, however, this data is the most comprehensive dataset of mental health problems in the UK general populations, therefore, was the most suitable dataset to use considering the aims of the study. Lastly, due to the cross-sectional nature of the data, it is not possible to conclude the directionality of the association between SES and alcohol use, including the indirect effect of social support and neighbourhood environment. Nonetheless, the current study provides some insight into the potential importance of decreased social support and living in a deprived neighbourhood environment, particularly among those from the lowest SES background who have a mental health problem using a nationally representative dataset.

### Conclusions

Individuals who met criteria for a mental health problem presented from a range of SES backgrounds. Compared to “professional occupation, degree-level educated, homeowners”, those of lower SES had increased odds of non-drinking. No associations between SES and harmful/probable dependence were found which contrasts with the alcohol harm paradox, however, when social support and neighbourhood environment were assessed, there were some associations among the most deprived SES group. Future research should examine the mechanisms of non-drinking and the role of social support and neighbourhood environment among individuals with a mental health problem.

## Supplementary Information

Below is the link to the electronic supplementary material.

Supplementary file1 (DOCX 185 KB)

## Data Availability

The 2014 APMS is a secure dataset held by NHS Digital, therefore, data from this manuscript cannot be made available. However, materials used to collect the data and published reports are publicly available at NHS Digital (https://digital.nhs.uk/data-and-information/publications/statistical/adult-psychiatric-morbidity-survey/adult-psychiatric-morbidity-survey-survey-of-mental-health-and-wellbeing-england-2014).

## References

[1] Glantz MD, Bharat C, Degenhardt L, Sampson NA, Scott KM, Lim CC et al (2020) The epidemiology of alcohol use disorders cross-nationally: findings from the World Mental Health surveys. Addict Behav 102:106128 31865172 10.1016/j.addbeh.2019.106128 PMC7416527

[2] Nutt DJ, King LA, Phillips LD (2010) Drug harms in the UK: a multicriteria decision analysis. The Lancet 376(9752):1558–1565 10.1016/S0140-6736(10)61462-6 21036393

[3] Puddephatt JA, Irizar P, Jones A, Gage SH, Goodwin L (2022) Associations of common mental disorder with alcohol use in the adult general population: a systematic review and meta-analysis. Addiction. 10.1111/add.15735 34729837 10.1111/add.15735 PMC9300028

[4] Grant BF, Goldstein RB, Saha TD, Chou SP, Jung J, Zhang H et al (2015) Epidemiology of DSM-5 alcohol use disorder: results from the national epidemiologic survey on alcohol and related conditions III. JAMA Psychiat 72(8):757–766 10.1001/jamapsychiatry.2015.0584 PMC5240584 26039070

[5] Whiteford HA, Degenhardt L, Rehm J, Baxter AJ, Ferrari AJ, Erskine HE et al (2013) Global burden of disease attributable to mental and substance use disorders: findings from the Global Burden of Disease Study 2010. The Lancet 382(9904):1575–1586 10.1016/S0140-6736(13)61611-6 23993280

[6] Rehm J, Shield KD (2019) Global burden of alcohol use disorders and alcohol liver disease. Biomedicines 7(4):99 31847084 10.3390/biomedicines7040099 PMC6966598

[7] Plana-Ripoll O, Pedersen CB, Agerbo E, Holtz Y, Erlangsen A, Canudas-Romo V et al (2019) A comprehensive analysis of mortality-related health metrics associated with mental disorders: a nationwide, register-based cohort study. The Lancet 394(10211):1827–1835 10.1016/S0140-6736(19)32316-5 31668728

[8] Walker ER, McGee RE, Druss BG (2015) Mortality in mental disorders and global disease burden implications: a systematic review and meta-analysis. JAMA Psychiat 72(4):334–341 10.1001/jamapsychiatry.2014.2502 PMC4461039 25671328

[9] Bellis MA, Hughes K, Nicholls J, Sheron N, Gilmore I, Jones L (2016) The alcohol harm paradox: using a national survey to explore how alcohol may disproportionately impact health in deprived individuals. BMC Public Health 16(1):1–10 26888538 10.1186/s12889-016-2766-x PMC4758164

[10] Mossakowski KN. 2014 Social causation and social selection. In: The Wiley Blackwell encyclopedia of health, illness, behavior, and society. Wiley, pp 2154-60

[11] Goldman N (1994) Social factors and health: the causation-selection issue revisited. Proc Natl Acad Sci 91(4):1251–1255 8108396 10.1073/pnas.91.4.1251 PMC43135

[12] Beard E, Brown J, West R, Angus C, Brennan A, Holmes J et al (2016) Deconstructing the alcohol harm paradox: a population based survey of adults in England. PLoS ONE 11(9):e0160666 27682619 10.1371/journal.pone.0160666 PMC5040414

[13] Beard E, Brown J, West R, Kaner E, Meier P, Michie S (2019) Associations between socio-economic factors and alcohol consumption: a population survey of adults in England. PLoS ONE 14(2):e0209442 30716098 10.1371/journal.pone.0209442 PMC6361426

[14] Katikireddi SV, Whitley E, Lewsey J, Gray L, Leyland AH (2017) Socioeconomic status as an effect modifier of alcohol consumption and harm: analysis of linked cohort data. Lancet Public Health 2(6):e267–e276 28626829 10.1016/S2468-2667(17)30078-6 PMC5463030

[15] Smith K, Foster J (2014) Alcohol, Health Inequalities and the Harm Paradox: Why some groups face greater problems despite consuming less alcohol. Institute of Alcohol Studies, London

[16] Puddephatt J-A, Jones A, Gage SH, Fear NT, Field M, McManus S et al (2021) Associations of alcohol use, mental health and socioeconomic status in England: Findings from a representative population survey. Drug Alcohol Depend 219:108463 33421804 10.1016/j.drugalcdep.2020.108463

[17] Marmot MG, Fuhrer R, Ettner SL, Marks NF, Bumpass LL, Ryff CD (1998) Contribution of psychosocial factors to socioeconomic differences in health. Milbank Q 76(3):403–448 9738169 10.1111/1468-0009.00097 PMC2751083

[18] Braveman PA, Cubbin C, Egerter S, Chideya S, Marchi KS, Metzler M et al (2005) Socioeconomic status in health researchone size does not fit all. JAMA 294(22):2879–2888 16352796 10.1001/jama.294.22.2879

[19] Boniface S, Lewer D, Hatch SL, Goodwin L (2020) Associations between interrelated dimensions of socio-economic status, higher risk drinking and mental health in South East London: a cross-sectional study. PLoS ONE 15(2):e0229093 32059050 10.1371/journal.pone.0229093 PMC7021306

[20] Taylor SE, Seeman TE (1999) Psychosocial resources and the SES-health relationship. Ann N Y Acad Sci 896(1):210–225 10681899 10.1111/j.1749-6632.1999.tb08117.x

[21] House JS, Umberson D, Landis KR (1988) Structures and processes of social support. Ann Rev Sociol 14(1):293–318

[22] Matthews KA, Gallo LC, Taylor SE (2010) Are psychosocial factors mediators of socioeconomic status and health connections? Ann N Y Acad Sci 1186(1):146–173 20201872 10.1111/j.1749-6632.2009.05332.x

[23] Cohen S, Wills TA (1985) Stress, social support, and the buffering hypothesis. Psychol Bull 98(2):310 3901065

[24] Taylor SE (2011) Social support: A review. The Oxford handbook of health psychology. Oxford University Press, New York, pp 189–214

[25] Smyth N, Siriwardhana C, Hotopf M, Hatch S (2015) Social networks, social support and psychiatric symptoms: social determinants and associations within a multicultural community population. Soc Psychiatry Psychiatr Epidemiol 50(7):1111–1120 25106666 10.1007/s00127-014-0943-8 PMC4464053

[26] Heerde JA, Hemphill SA (2018) Examination of associations between informal help-seeking behavior, social support, and adolescent psychosocial outcomes: a meta-analysis. Dev Rev 47:44–62

[27] Jarnecke AM, South SC (2014) Genetic and environmental influences on alcohol use problems: moderation by romantic partner support, but not family or friend support. Alcohol Clin Exp Res 38(2):367–75 24164253 10.1111/acer.12263

[28] Sacco P, Bucholz KK, Harrington D (2014) Gender differences in stressful life events, social support, perceived stress, and alcohol use among older adults: results from a national survey. Subst Use Misuse 49(4):456–465 24131262 10.3109/10826084.2013.846379 PMC4729187

[29] Eddie D, Hoffman L, Vilsaint C, Abry A, Bergman B, Hoeppner B et al (2019) Lived experience in new models of care for substance use disorder: a systematic review of peer recovery support services and recovery coaching. Front Psychol. 10.3389/fpsyg.2019.01052 31263434 10.3389/fpsyg.2019.01052 PMC6585590

[30] Stillman MA, Sutcliff J (2020) Predictors of relapse in alcohol use disorder: identifying individuals most vulnerable to relapse. Addctn Subst Abus 1(1):3–8

[31] McElroy E, McIntyre JC, Bentall RP, Wilson T, Holt K, Kullu C et al (2019) Mental health, deprivation, and the neighborhood social environment: a network analysis. Clin Psychol Sci. 10.1177/2167702619830640

[32] Businelle MS, Kendzor DE, Reitzel LR, Costello TJ, Cofta-Woerpel L, Li Y et al (2010) Mechanisms linking socioeconomic status to smoking cessation: a structural equation modeling approach. Health Psychol 29(3):262–273 20496980 10.1037/a0019285 PMC2922845

[33] Fone DL, Farewell DM, White J, Lyons RA, Dunstan FD (2013) Socioeconomic patterning of excess alcohol consumption and binge drinking: a cross-sectional study of multilevel associations with neighbourhood deprivation. BMJ Open 3(4):e002337 23587771 10.1136/bmjopen-2012-002337 PMC3641461

[34] Karriker-Jaffe KJ, Liu H, Kaplan LM (2016) Understanding associations between neighborhood socioeconomic status and negative consequences of drinking: a moderated mediation analysis. Prev sci 17(4):513–24 26898509 10.1007/s11121-016-0641-8 PMC5031144

[35] Karriker-Jaffe KJ (2011) Areas of disadvantage: a systematic review of effects of area-level socioeconomic status on substance use outcomes. Drug Alcohol Rev 30(1):84–95 21219502 10.1111/j.1465-3362.2010.00191.x PMC3057656

[36] Phelan JC, Link BG (2013) Fundamental cause theory. Medical sociology on the move: New directions in theory. Springer, pp 105–25

[37] McManus S, Bebbington PE, Jenkins R, Morgan Z, Brown L, Collinson D et al (2020) Data resource profile: adult psychiatric morbidity survey (APMS). Int J Epidemiol 49(2):361–362 31725160 10.1093/ije/dyz224

[38] World Health Organization. 2001 AUDIT: The alcohol use disorders identification test: Guidelines for use in primary health care: World Health Organization, pp 1–40

[39] Meneses-Gaya C, Zuardi AW, Loureiro SR, Hallak JE, Trzesniak C, de Azevedo Marques JM et al (2010) Is the full version of the AUDIT really necessary study of the validity and internal construct of its abbreviated versions. Alcohol Clin Exp Res 34(8):1417–24 20491736 10.1111/j.1530-0277.2010.01225.x

[40] Cox B, Blaxter M, Buckle A, Fenner N, Golding J, Gore M et al (1987) The Health and Lifestyle Survey Preliminary report of a nationwide survey of the physical and mental health, attitudes and lifestyle of a random sample of 9,003 British adults. Health Promotion Research Trust

[41] McManus S, Bebbington PE, Jenkins R, Brugha T (2016) Mental health and wellbeing in England: the adult psychiatric morbidity survey 2014. NHS digital

[42] Brugha T, Bebbington P, MacCarthy B, Potter J, Sturt E, Wykes T (1987) Social networks, social support and the type of depressive illness. Acta Psychiatr Scand 76(6):664–673 3442257 10.1111/j.1600-0447.1987.tb02937.x

[43] Brugha TS, Sturt E, MacCarthy B, Potter J, Wykes T, Bebbington P (1987) The Interview measure of social relationships: the description and evaluation of a survey instrument for assessing personal social resources. Soc Psychiatry 22:123–128 3589784 10.1007/BF00584017

[44] Kawachi I, Berkman L (2000) Social cohesion, social capital, and health. Soc epidemiol 174(7):290–319

[45] Mouratidis K (2020) Neighborhood characteristics, neighborhood satisfaction, and well-being: The links with neighborhood deprivation. Land Use Policy 99:104886

[46] Muthén L (2007) Mplus user guide. Muthén & Muthén. Inc, California

[47] Corraini P, Olsen M, Pedersen L, Dekkers OM, Vandenbroucke JP (2017) Effect modification, interaction and mediation: an overview of theoretical insights for clinical investigators. Clin Epidemiol 9:331–338 28652815 10.2147/CLEP.S129728 PMC5476432

[48] Feingold A (2019) New approaches for estimation of effect sizes and their confidence intervals for treatment effects from randomized controlled trials. Quant method Psychol 15(2):96 32775313 10.20982/tqmp.15.2.p096 PMC7413603

[49] World Health Organization. 2017.Depression and other common mental disorders: global health estimates. World Health Organization, pp 1–21

[50] Hardoon S, Hayes JF, Blackburn R, Petersen I, Walters K, Nazareth I et al (2013) Recording of severe mental illness in United Kingdom primary care, 2000–2010. PLoS ONE 8(12):e82365 24349267 10.1371/journal.pone.0082365 PMC3861391

[51] Azur MJ, Stuart EA, Frangakis C, Leaf PJ (2011) Multiple imputation by chained equations: what is it and how does it work? Int J Methods Psychiatr Res 20(1):40–49 21499542 10.1002/mpr.329 PMC3074241

[52] Sterne JA, White IR, Carlin JB, Spratt M, Royston P, Kenward MG et al (2009) Multiple imputation for missing data in epidemiological and clinical research: potential and pitfalls. BMJ 338:b2393 19564179 10.1136/bmj.b2393 PMC2714692

[53] Agerbo E, Sullivan PF, Vilhjálmsson BJ, Pedersen CB, Mors O, Børglum AD et al (2015) Polygenic risk score, parental socioeconomic status, family history of psychiatric disorders, and the risk for schizophrenia: a Danish population-based study and meta-analysis. JAMA Psychiat 72(7):635–641 10.1001/jamapsychiatry.2015.0346 25830477

[54] Smith DJ, Nicholl BI, Cullen B, Martin D, Ul-Haq Z, Evans J et al (2013) Prevalence and characteristics of probable major depression and bipolar disorder within UK Biobank: cross-sectional study of 172,751 participants. PLoS ONE 8(11):e75362 24282498 10.1371/journal.pone.0075362 PMC3839907

[55] Skogen JC, Harvey SB, Henderson M, Stordal E, Mykletun A (2009) Anxiety and depression among abstainers and low-level alcohol consumers the Nord-Trøndelag health study. Addiction 104(9):1519–1529 19686521 10.1111/j.1360-0443.2009.02659.x

[56] Stockwell T, Zhao J, Panwar S, Roemer A, Naimi T, Chikritzhs T (2016) Do, “moderate” drinkers have reduced mortality risk? a systematic review and meta-analysis of alcohol consumption and all-cause mortality. J Stud Alcohol Drugs 77(2):185–198 26997174 10.15288/jsad.2016.77.185 PMC4803651

[57] Collins SE (2016) Associations between socioeconomic factors and alcohol outcomes. Alcohol Res 38(1):83–94 27159815 10.35946/arcr.v38.1.11 PMC4872618

[58] Matheson FI, White HL, Moineddin R, Dunn JR, Glazier RH (2012) Drinking in context: the influence of gender and neighbourhood deprivation on alcohol consumption. J Epidemiol Community Health. 10.1136/jech.2010.112441 21330461 10.1136/jech.2010.112441

[59] Kuipers MAG, van Poppel MNM, van den Brink W, Wingen M, Kunst AE (2012) The association between neighborhood disorder, social cohesion and hazardous alcohol use: a national multilevel study. Drug Alcohol Depend 126(1):27–34 22572208 10.1016/j.drugalcdep.2012.04.008

