# Supplementary material for: Socioeconomic status, alcohol use and the role of social support and neighbourhood environment among individuals meeting criteria for a mental health problem: a cross-sectional study
Source: Soc Psychiatry Psychiatr Epidemiol. 2024 Apr 26;59(12):2177–88. doi: 10.1007/s00127-024-02670-w (PMC11522183; doi:10.1007/s00127-024-02670-w)
Supplement: Supplementary file 1 — Supplementary file1 (DOCX 185 KB) [file 127_2024_2670_MOESM1_ESM.docx]

Supplementary materials

Table S1: An overview of mental health problems included in the study and the measures used to assess criteria

| Type of mental health problem | Measure used |
| --- | --- |
| Depression (including mild, moderate, and severe) | Clinical Interview Schedule-Revised (CISR) |
| Anxiety (including generalized anxiety disorder, obsessive compulsive disorder, and panic disorder) | CISR |
| Phobia (including social, specific and agoraphobia) | CISR |
| Post-Traumatic Stress Disorder | PTSD Checklist-Civilian |
| Bipolar Disorder | Mood Disorder Questionnaire |
| Anti-Social Personality Disorder | Structured Clinical Interview for DSM-IV Personality Disorders (SCID-II) |
| Borderline Personality Disorder | SCID-II |
| Probable Psychotic Disorder | Psychosis Screening Questionnaire and self-reported hospital stays |
| Attention Deficit Hyperactivity Disorder | Adult ADHD Self-Report Scale-v1.1 |

Table S2: Sample characteristics of the 2014 APMS sample stratified by meeting criteria for a mental health problem

|  |  | Has mental health problem (N=1,463) | Does not have a mental health problem (N=6,083) |
| --- | --- | --- | --- |
| Gender | Male | 576 (49.31) | 2,482 (48.73) |
|  | Female | 887 (50.69) | 3,601 (51.27) |
| Age | 16-34 | 422 (41.06) | 1,173 (28.48) |
|  | 35-54 | 574 (35.55) | 1,902 (32.97) |
|  | 55-74 | 384 (19.52) | 2,031 (27.10) |
|  | 75+ | 85 (3.87) | 977 (11.45) |
| Ethnicity | White | 1,294 (86.36) | 5,519 (87.58) |
|  | Non-white | 158 (13.64) | 547 (12.42) |
| Social occupational grade | Managerial/professional | 300 (21.56) | 1,495 (26.85) |
|  | Intermediate | 139 (9.70) | 539 (8.95) |
|  | Lower supervisory | 85 (6.15) | 341 (6.28) |
|  | Student | 50 (6.78) | 96 (3.11) |
|  | Retired | 245 (11.46) | 2,022 (24.50) |
|  | Never worked/not worked in the past year | 628 (43.39) | 1,559 (29.78) |
| In receipt of any out of work benefits | No | 1,105 (81.37) | 5,739 (95.57) |
|  | Yes | 644 (18.63) | 306 (4.43) |
| In debt | No | 1,162 (83.35) | 5,766 (95.27) |
|  | Yes | 277 (16.65) | 287 (4.73) |
| Education | Degree or higher | 395 (27.45) | 2,015 (34.73) |
|  | A-Level/GCSE | 654 (49.90) | 2,285 (41.59) |
|  | Foreign qualifications | 35 (2.30) | 237 (3.42) |
|  | No qualifications | 359 (20.62) | 1,484 (20.26) |
| Housing tenure | Homeowner | 695 (49.11) | 4,226 (67.65) |
|  | Private renter | 421 (26.69) | 849 (13.33) |
|  | Social renter | 331 (24.20) | 973 (19.02) |
| Household composition | Lives alone, without children | 475 (17.75) | 1,720 (14.94) |
|  | Lives with another adult, without children | 424 (30.14) | 2,308 (38.26) |
|  | Lives in a family | 345 (22.35) | 1,231 (21.25) |
|  | Lives in a large adult household | 219 (29.75) | 824 (25.54) |

#### Table S3: Model fit from one- to six-class model

| Fit indices | Model replicated | Loglikelihood | No. of parameters | LMR-LRT | *p* | AIC | BIC | SSABIC | Entropy |
| --- | --- | --- | --- | --- | --- | --- | --- | --- | --- |
| One-class | NA | -11366.50 | 23 | NA | NA | 22779.01 | 22900.64 | 22827.57 | NA |
| Two-class | Yes | -8368.92 | 35 | 1134.54 | **0.01** | 16807.83 | 16992.92 | 16881.73 | 0.93 |
| Three-class | Yes | -7861.52 | 55 | 1008.77 | 0.61 | 15833.04 | 16123.89 | 15949.18 | **0.88** |
| Four-class | Yes | -7703.43 | 75 | 314.31 | 0.75 | 15556.86 | **15953.48** | **15715.23** | 0.81 |
| Five-class | Yes | -7617.33 | 95 | 198.77 | 0.27 | **15424.67** | 15927.05 | 15625.27 | 0.86 |
| Six-class | Yes | **-7550.69** | 115 | 132.63 | 0.63 | 15331.39 | 15939.54 | 15574.22 | 0.83 |

*Bold in-table text indicates the best-fitting class according to the model-fit indicator

Table S4: Class probabilities for the individual SES indicators

| Variable | Category | Class one |  | Class two |  | Class three |  | Class four |  |
| --- | --- | --- | --- | --- | --- | --- | --- | --- | --- |
|  |  | Probability | p | Probability | p | Probability | p | Probability | p |
| Social occupational grade | Managerial/professional | **0.03** | **0.04** | 0.07 | 0.05 | 0.01 | 0.80 | **0.76** | **0.01** |
|  | Intermediate/small employers/own account worker | **0.03** | **0.01** | **0.24** | **0.01** | **0.05** | **0.03** | **0.19** | **0.01** |
|  | Lower supervisory/technical/routine/semi-routine | **0.13** | **0.01** | **0.42** | **0.01** | **0.09** | **0.01** | 0.04 | 0.28 |
|  | Student | 0.01 | 0.16 | **0.16** | **0.01** | 0.00 | 1.00 | 0.00 | 1.00 |
|  | Retired | 0.00 | 1.00 | 0.00 | 1.00 | **0.76** | **0.01** | 0.00 | 1.00 |
|  | Never/not worked in the past year | **0.80** | **0.01** | **0.11** | **0.01** | **0.10** | **0.01** | 0.01 | 0.48 |
| In debt | No | **0.58** | **0.01** | **0.84** | **0.01** | **0.95** | **0.01** | **0.95** | **0.01** |
|  | Yes | **0.42** | **0.01** | **0.16** | **0.01** | **0.05** | **0.01** | **0.05** | **0.01** |
| Receiving any out of work benefits | No | **0.16** | **0.01** | **0.96** | **0.01** | **0.95** | **0.01** | **0.99** | **0.01** |
|  | Yes | **0.84** | **0.01** | **0.04** | **0.01** | **0.05** | **0.01** | 0.01 | 0.15 |
| Educational attainment | Degree or higher | **0.10** | **0.01** | **0.15** | **0.01** | **0.21** | **0.01** | **0.67** | **0.01** |
|  | A-Level/GCSE | **0.46** | **0.01** | **0.72** | **0.01** | **0.26** | **0.01** | **0.29** | **0.01** |
|  | Other | 0.01 | 0.10 | 0.01 | 0.23 | **0.09** | **0.01** | 0.01 | 0.06 |
|  | None | **0.43** | **0.01** | **0.12** | **0.01** | **0.44** | **0.01** | 0.03 | 0.24 |
| Housing tenure | Homeowner | **0.12** | **0.01** | **0.46** | **0.01** | **0.72** | **0.01** | **0.70** | **0.01** |
|  | Social renter | **0.64** | **0.01** | **0.23** | **0.01** | **0.24** | **0.01** | **0.05** | **0.01** |
|  | Private renter | **0.25** | **0.01** | **0.31** | **0.01** | **0.05** | **0.01** | **0.25** | **0.01** |
|  |  | Mean (SE) | p | Mean (SE) | p | Mean (SE) | p | Mean (SE) | p |
| Household type | Lives alone, without children | Ref | Ref | Ref | Ref | Ref | Ref | Ref | Ref |
|  | Lives with another adult, without children | **1.01 (0.28)** | **0.01** | **-2.49 (0.36)** | **0.01** | **0.95 (0.29)** | **0.01** | -0.31 (0.34) | 0.36 |
|  | Family | 0.24 (0.31) | 0.44 | **-0.85 (0.17)** | **0.01** | **1.43 (0.28)** | **0.01** | **0.08 (0.32)** | **0.01** |
|  | Large adult household | 0.56 (0.30) | 0.06 | **-0.66 (0.16)** | **0.01** | -15.00 (0.00) | 999.00 | 0.45 (0.34) | 0.19 |

*Bold indicates significance

Table S5: A sensitivity analysis showing the prevalence of the latent SES classes by type of mental health problem

|  |  | Class one: *“Economically inactive, GCSE-level educated or lower, social renters”* (n=341, 19.25%) | Class two: *“Routine/intermediate occupations, GCSE-level educated, mixed owner/renters”* (n=440, 42.24%) | Class three: *“Retired, no formal education, homeowners”* (n=311, 16.31%) | Class four: *“Professional occupation, degree-level educated, homeowners”* (n=339, 22.20%) |
| --- | --- | --- | --- | --- | --- |
| Type of mental health problem |  | *N* (weighted %) | *N* (weighted %) | *N* (weighted %) | *N* (weighted %) |
| CMD | Yes | 216 (25.42) | 195 (36.03) | 164 (18.94) | 143 (19.61) |
|  | No | 125 (13.84) | 245 (47.68) | 147 (14.01) | 196 (24.47) |
| SMI | Yes | 97 (42.87) | 48 (34.78) | 22 (8.56) | 28 (13.79) |
|  | No | 244 (15.67) | 392 (43.37) | 289 (17.49) | 311 (23.47) |

**CMD=Common mental disorder (including depression and anxiety disorders), SMI=Severe mental illness (including bipolar disorder, probable psychotic disorder and any other psychotic disorder)*

Table S6: Mean scores and standard deviations of social support and neighbourhood disadvantage scores stratified by SES classes and alcohol use categories, respectively

|  |  | Social support (*n*=1,436) | Neighbourhood disadvantage (*n*=1,436) |
| --- | --- | --- | --- |
|  |  | M (SD) | M (SD) |
| SES classes | **Class 1 – *Economically inactive, GCSE-level educated or lower, social renters*** | 17.97 (3.76) | 23.65 (7.66) |
|  | **Class 2 - Routine/intermediate occupations, GCSE-level educated, mixed owner/renters** | 19.71 (2.41) | 21.16 (7.51) |
|  | **Class 3 – *Retired, no formal education, homeowners*** | 19.41 (2.84) | 18.00 (6.51) |
|  | **Class 4 – *Professional occupation, degree-level educated, homeowners*** | 19.98 (2.01) | 19.47 (6.70) |
| Alcohol use | **Non-drinker** | 18.58 (3.52) | 21.48 (7.93) |
|  | **Low-risk drinker** | 19.69 (2.42) | 20.05 (7.11) |
|  | **Hazardous drinker** | 19.60 (2.63) | 20.24 (7.17) |
|  | **Harmful drinker** | 18.66 (3.35) | 22.25 (7.28) |

#### **Sensitivity analyses**

*Latent class analysis with household income and imputed values*

A latent analysis with household income as an additional SES variable and with imputed values was conducted from a one-class to six-class model. Inspection of AIC, BIC SSABIC, and entropy values indicated that three-, four-, five-, or six-class models were viable (see table s7). The biggest drop in BIC and SSABIC values were observed from a three- to four-class model (see table s7). Inspection of class characteristics for three and four-class models suggest that a three-class model was defined by social occupational grade (economically inactive, economically active, and retired groups) whereas a four-class model was defined by multiple indicators of SES. Taken together, a four-class model was deemed a viable model. Compared with the original latent class model (non-imputed values and without household income as an SES indicator), inspection of class characteristics indicated the same as that of the original model (without household income). Inclusion of household income only confirmed that the majority of “economically inactive, social renters” were on the lowest income, the majority of “professional occupation, degree-level educated, homeowners” were on the highest incomes, while over 70% of “routine/intermediate occupation, GCSE-Level educated, mixed owner/renters” were on middle to lower income, and half of “retired, no formal education, homeowners” were on lower income (see table s8).

#### Table S7: Post hoc model fit from one- to six-class model with household income added to the model

| Fit indices | Model replicated | Loglikelihood | No. of parameters | LMR-LRT | *p* | AIC | BIC | SSABIC | Entropy |
| --- | --- | --- | --- | --- | --- | --- | --- | --- | --- |
| One-class | NA | -11684.18 | 27 | NA | NA | 27422.36 | 27565.14 | 27479.37 | NA |
| Two-class | Yes | -10573.18 | 43 | 1361.53 | 0.16 | 21232.35 | 21459.75 | 21323.15 | 0.74 |
| Three-class | Yes | -10021.97 | 67 | 1308.04 | 0.25 | 20177.94 | 20532.25 | 20319.41 | **0.86** |
| Four-class | Yes | -9786.38 | 91 | 468.87 | 0.76 | 19754.77 | 20235.998 | 19946.92 | 0.82 |
| Five-class | Yes | -9696.42 | 115 | 179.09 | 0.74 | 19622.84 | **20230.98** | 19865.66 | 0.81 |
| Six-class | Yes | -9621.70 | 139 | 148.75 | 0.56 | **19521.41** | 20256.47 | **19814.91** | 0.81 |

Table S8: Post hoc class probabilities for the individual SES indicators

| Variable | Category | Class one |  | Class two |  | Class three |  | Class four |  |
| --- | --- | --- | --- | --- | --- | --- | --- | --- | --- |
|  |  | Probability | p | Probability | p | Probability | p | Probability | p |
| Social occupational grade | Managerial/professional | **0.03** | **0.03** | **0.10** | **0.01** | 0.00 | 1.00 | **0.68** | **0.01** |
|  | Intermediate/small employers/own account worker | **0.05** | **0.03** | **0.23** | **0.01** | **0.05** | **0.01** | **0.21** | **0.01** |
|  | Lower supervisory/technical/routine/semi-routine | **0.14** | **0.01** | **0.40** | **0.01** | **0.08** | **0.01** | **0.10** | **0.03** |
|  | Student | 0.01 | 0.32 | **0.17** | **0.01** | 0.00 | 1.00 | 0.00 | 1.00 |
|  | Retired | 0.00 | 1.00 | 0.00 | 1.00 | **0.76** | **0.01** | 0.00 | 1.00 |
|  | Never/not worked in the past year | **0.78** | **0.01** | **0.11** | **0.01** | **0.11** | **0.01** | 0.01 | 0.12 |
| In debt | No | **0.58** | **0.01** | **0.84** | **0.01** | **0.95** | **0.01** | **0.95** | **0.01** |
|  | Yes | **0.42** | **0.01** | **0.16** | **0.01** | **0.05** | **0.01** | **0.05** | **0.01** |
| Receiving any out of work benefits | No | **0.20** | **0.01** | **0.95** | **0.01** | **0.95** | **0.01** | **0.99** | **0.01** |
|  | Yes | **0.80** | **0.01** | **0.05** | **0.01** | **0.05** | **0.01** | 0.01 | 0.35 |
| Household income | Less than £12,999 | **0.65** | **0.01** | **0.24** | **0.01** | **0.30** | **0.01** | 0.03 | 0.06 |
|  | More than or equal to £12,999-less than £20,279 | **0.18** | **0.01** | **0.29** | **0.01** | **0.27** | **0.01** | 0.04 | 0.01 |
|  | More than or equal to £20,279-less than £31,666 | **0.09** | **0.01** | **0.25** | **0.01** | **0.25** | **0.01** | **0.20** | **0.01** |
|  | More than or equal to £31,666-less than £52,499 | **0.05** | **0.01** | **0.11** | **0.01** | **0.11** | **0.01** | **0.29** | **0.01** |
|  | More than or equal to £52,499 | **0.03** | **0.03** | **0.11** | **0.01** | **0.06** | **0.01** | **0.44** | **0.01** |
| Educational attainment | Degree or higher | **0.10** | **0.01** | **0.17** | **0.01** | **0.21** | **0.01** | **0.62** | **0.01** |
|  | A-Level/GCSE | **0.47** | **0.01** | **0.73** | **0.01** | **0.26** | **0.01** | **0.31** | **0.01** |
|  | Other | 0.01 | 0.94 | 0.01 | 0.44 | **0.09** | **0.01** | 0.02 | 0.05 |
|  | None | **0.43** | **0.01** | **0.10** | **0.01** | **0.44** | **0.01** | **0.06** | **0.01** |
| Housing tenure | Homeowner | **0.11** | **0.01** | **0.43** | **0.01** | **0.71** | **0.01** | **0.75** | **0.14** |
|  | Social renter | **0.64** | **0.01** | **0.24** | **0.01** | **0.24** | **0.01** | 0.02 | 0.14 |
|  | Private renter | **0.25** | **0.01** | **0.32** | **0.01** | **0.05** | **0.01** | **0.23** | **0.01** |
|  |  | Mean (SE) | p | Mean (SE) | p | Mean (SE) | p | Mean (SE) | p |
| Household type | Lives alone, without children | Ref | Ref | Ref | Ref | Ref | Ref | Ref | Ref |
|  | Lives with another adult, without children | **0.94 (0.27)** | **0.01** | **-2.68 (0.43)** | **0.01** | **0.89 (0.27)** | **0.01** | -0.35 (0.35) | 0.31 |
|  | Family | 0.12 (0.30) | 0.70 | **-0.92 (0.18)** | **0.01** | **1.35 (0.27)** | **0.01** | **0.81 (0.33)** | **0.01** |
|  | Large adult household | 0.53 (0.28) | 0.06 | **-0.63 (0.19)** | **0.01** | -15.00 (0.00) | 999.00 | 0.32 (0.34) | 0.36 |

*Association between latent SES model (household income included in model with imputed values) and alcohol use*

As with the original model, compared to “professional occupation, degree-level educated, homeowners”, all other SES groups were two and four times more likely to be non-drinkers (see table S9). Compared to “professional occupation, degree-level educated, homeowners”, “retired, no formal education, homeowners” were less likely to hazardous drinkers (OR=0.34, 95% CI=0.22-0.53). Compared to “professional managerial, degree-level educated, homeowners”, “retired, no formal education, homeowners” were less likely to harmful drinkers (OR=0.39, 95% CI=0.21-0.75, see table s9).

Table S9: Post hoc weighted associations of classes of SES (including household income) and alcohol use

|  | Non-drinker | | | Low-risk use (reference group) | Hazardous use | | | Harmful/probable dependence | | |
| --- | --- | --- | --- | --- | --- | --- | --- | --- | --- | --- |
|  | *n* (weighted %) | MOR (95% CI) | *p* | *n* (weighted %) | *n* (weighted %) | MOR (95% CI) | *p* | *n* (weighted %) | MOR (95% CI) | *p* |
| Class 1 – *Economically inactive, GCSE-level or lower educated, social renters* | 130 (29.92) | **4.58 (3.12-6.73)** | **0.01** | 120 (14.72) | 54 (12.79) | 0.74 (0.50-1.09) | 0.28 | 33 (23.90) | 1.59 (0.93-2.71) | 0.08 |
| Class 2 - *Routine/intermediate occupation, GCSE-level educated, mixed owner/renters* | 85 (35.89) | **1.97 (1.34-2.89)** | **0.01** | 204 (41.05) | 91 (47.94) | 1.00 (0.70-1.41) | 0.57 | 35 (41.08) | 0.98 (0.58-1.65) | 0.55 |
| Class 3 – *Retired, no formal education, homeowner* | 105 (21.80) | **3.01 (2.07-4.39)** | **0.01** | 147 (16.30) | 29 (6.49) | **0.34 (0.22-0.53)** | **0.01** | 12 (6.53) | **0.39 (0.21-0.75)** | 0.11 |
| Class 4 - *Professional occupation, degree-level educated, homeowner s* | 44 (12.40) | Ref. | Ref. | 195 (27.93) | 87 (32.77) | Ref. | Ref. | 29 (28.50) | Ref. | Ref. |

*The indirect effect of social support and neighbourhood environment on associations between latent SES groups (with household income and imputed values) and alcohol use*

The indirect effect of social support and neighbourhood environment remained the same when using the four-class model with imputed values and household income as an additional indicator. There was a positive indirect effect of social support on the association between “economically inactive, GCSE-level or lower educated, social renters”, and “retired, no formal education, homeowners”, with being a non-drinker, and harmful drinker, respectively (see table s10). There was a positive indirect effect of neighbourhood environment on the association between “economically inactive, GCSE-Level or lower educated, social renters” and harmful drinker (see table s10).

Table S10: Post hoc analysis of the indirect effect of associations between SES (including household income) and alcohol use via social support and neighbourhood environment

|  |  | **Social support (*n*=1,436)** | | | **Neighbourhood environment (*n*=1,436)** | | |
| --- | --- | --- | --- | --- | --- | --- | --- |
| **Class 1 – *Economically inactive, GCSE-level or lower educated, social renters*** |  | **Unstandardised coefficient (SE)** | **95% CI** | ***p*** | **Unstandardised coefficient (SE)** | **95% CI** | ***p*** |
|  | Non-drinker | **0.20 (0.07)** | **0.09-0.31** | **0.01** | 0.05 (0.04) | -0.02-0.12 | 0.27 |
|  | Low-risk drinker | Ref. | Ref. | Ref. | Ref. | Ref. | Ref. |
|  | Hazardous drinker | 0.03 (0.07) | -0.10-0.15 | 0.72 | 0.01 (0.05) | -0.07-0.09 | 0.84 |
|  | Harmful/probable dependent drinker | **0.27 (0.10)** | **0.11-0.44** | **0.01** | **0.13 (0.06)** | **0.04-0.22** | **0.02** |
| **Class 2 - R*outine/intermediate occupation, GCSE-level educated, mixed owner/renters*** |  | **Unstandardised coefficient (SE)** | **95% CI** | ***p*** | **Unstandardised coefficient (SE)** | **95% CI** | ***p*** |
|  | Non-drinker | 0.03 (0.02) | 0.00-0.05 | 0.13 | 0.03 (0.02) | -0.01-0.06 | 0.29 |
|  | Low-risk use | Ref. | Ref. | Ref. | Ref. | Ref. | Ref. |
|  | Hazardous use | 0.00 (0.01) | -0.01-0.02 | 0.73 | 0.01 (0.02) | -0.04-0.05 | 0.84 |
|  | Harmful/probable dependence | 0.04 (0.02) | -0.01-0.07 | 0.15 | 0.07 (0.03) | 0.01-0.12 | 0.05 |
| **Class 3 – *Retired, no formal educated, homeowners*** |  | **Unstandardised coefficient (SE)** | **95% CI** | ***p*** | **Unstandardised coefficient (SE)** | **95% CI** | ***p*** |
|  | Non-drinker | **0.05 (0.02)** | **0.01-0.09** | **0.02** | -0.02 (0.02) | -0.06-0.01 | 0.36 |
|  | Low-risk use | Ref. | Ref. | Ref. | Ref. | Ref. | Ref. |
|  | Hazardous use | 0.01 (0.02) | -0.02-0.04 | 0.72 | -0.00 (0.02) | -0.04-0.03 | 0.84 |
|  | Harmful/probable dependence | **0.07 (0.03)** | **0.02-0.12** | **0.03** | -0.06 (0.03) | -0.11- -0.01 | 0.06 |
| **Class 4 – *Professional occupation, degree-level educated, homeowners*** |  | Ref. | Ref. | Ref. | Ref. | Ref. | Ref. |


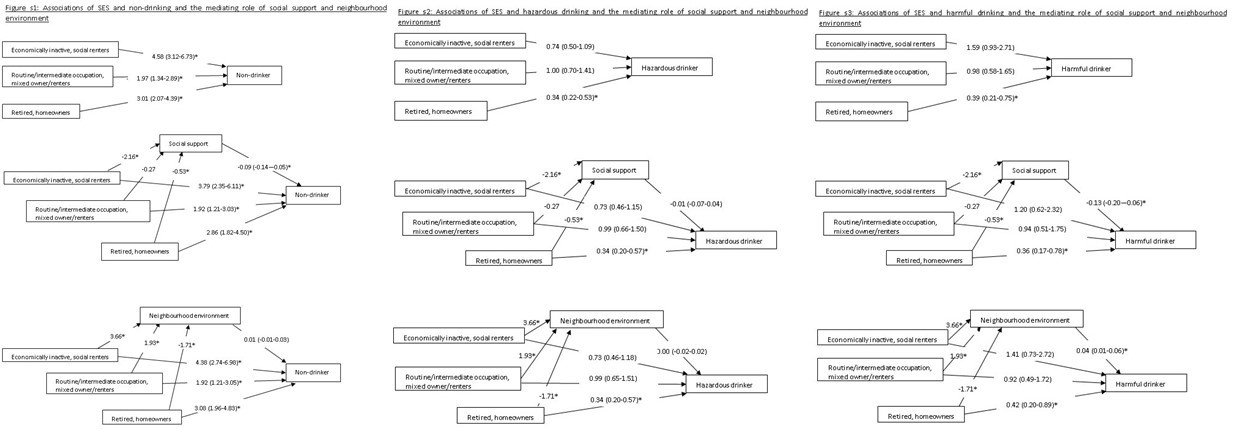


## Table S11: STROBE Checklist

STROBE Statement—Checklist of items that should be included in reports of ***cross-sectional studies***

|  | Item No | Recommendation | Page No |
| --- | --- | --- | --- |
| **Title and abstract** | 1 | (*a*) Indicate the study’s design with a commonly used term in the title or the abstract | 1 |
|  |  | (*b*) Provide in the abstract an informative and balanced summary of what was done and what was found | 1-2 |
| Introduction | | | |
| Background/rationale | 2 | Explain the scientific background and rationale for the investigation being reported | 2-3 |
| Objectives | 3 | State specific objectives, including any prespecified hypotheses | 3 |
| Methods | | | |
| Study design | 4 | Present key elements of study design early in the paper | 4 |
| Setting | 5 | Describe the setting, locations, and relevant dates, including periods of recruitment, exposure, follow-up, and data collection | 4 |
| Participants | 6 | (*a*) Give the eligibility criteria, and the sources and methods of selection of participants | 5 |
| Variables | 7 | Clearly define all outcomes, exposures, predictors, potential confounders, and effect modifiers. Give diagnostic criteria, if applicable | 4-5 |
| Data sources/ measurement | 8* | For each variable of interest, give sources of data and details of methods of assessment (measurement). Describe comparability of assessment methods if there is more than one group | 4-5 |
| Bias | 9 | Describe any efforts to address potential sources of bias | 5-6 |
| Study size | 10 | Explain how the study size was arrived at | 5 |
| Quantitative variables | 11 | Explain how quantitative variables were handled in the analyses. If applicable, describe which groupings were chosen and why | 4-6 |
| Statistical methods | 12 | (*a*) Describe all statistical methods, including those used to control for confounding | 5-8 and figure 1 |
|  |  | (*b*) Describe any methods used to examine subgroups and interactions | 8 |
|  |  | (*c*) Explain how missing data were addressed | 4-5 |
|  |  | (*d*) If applicable, describe analytical methods taking account of sampling strategy | 4-5 and figure 1 |
|  |  | (*e*) Describe any sensitivity analyses | 8 |
| Results | | | |
| Participants | 13* | (a) Report numbers of individuals at each stage of study—eg numbers potentially eligible, examined for eligibility, confirmed eligible, included in the study, completing follow-up, and analysed | 4-8 and figure 1 |
|  |  | (b) Give reasons for non-participation at each stage | 4-8 and figure 1 |
|  |  | (c) Consider use of a flow diagram | Figure 1 |
| Descriptive data | 14* | (a) Give characteristics of study participants (eg demographic, clinical, social) and information on exposures and potential confounders | 8-9, Table 1 |
|  |  | (b) Indicate number of participants with missing data for each variable of interest | 4-5 |
| Outcome data | 15* | Report numbers of outcome events or summary measures | 8-15, Tables 1, 2 and 3 |
| Main results | 16 | (*a*) Give unadjusted estimates and, if applicable, confounder-adjusted estimates and their precision (eg, 95% confidence interval). Make clear which confounders were adjusted for and why they were included | 8-15, Tables 1, 2 and 3 |
|  |  | (*b*) Report category boundaries when continuous variables were categorized | 4-5 |
|  |  | (*c*) If relevant, consider translating estimates of relative risk into absolute risk for a meaningful time period | NA |
| Other analyses | 17 | Report other analyses done—eg analyses of subgroups and interactions, and sensitivity analyses | 8 and supplementary materials |
| Discussion | | | |
| Key results | 18 | Summarise key results with reference to study objectives | 16-17 |
| Limitations | 19 | Discuss limitations of the study, taking into account sources of potential bias or imprecision. Discuss both direction and magnitude of any potential bias | 17-18 |
| Interpretation | 20 | Give a cautious overall interpretation of results considering objectives, limitations, multiplicity of analyses, results from similar studies, and other relevant evidence | 10-12 |
| Generalisability | 21 | Discuss the generalisability (external validity) of the study results | 9-12 |
| Other information | | | |
| Funding | 22 | Give the source of funding and the role of the funders for the present study and, if applicable, for the original study on which the present article is based | 14 |

*Give information separately for exposed and unexposed groups.

**Note:** An Explanation and Elaboration article discusses each checklist item and gives methodological background and published examples of transparent reporting. The STROBE checklist is best used in conjunction with this article (freely available on the Web sites of PLoS Medicine at http://www.plosmedicine.org/, Annals of Internal Medicine at http://www.annals.org/, and Epidemiology at http://www.epidem.com/). Information on the STROBE Initiative is available at www.strobe-statement.org.
